# Risk Factors for COVID-19 Mortality Among People Living with HIV: A Scoping Review

**DOI:** 10.1007/s10461-022-03578-9

**Published:** 2022-01-13

**Authors:** Karan Varshney, Prerana Ghosh, Helena Stiles, Rosemary Iriowen

**Affiliations:** 1 College of Population Health, Thomas Jefferson University, Philadelphia, PA USA; 2 School of Medicine, Deakin University, 75 Pigdons Rd, Waurn Ponds, VIC 3216 Australia

**Keywords:** COVID-19, SARS-COV-2, HIV, Mortality, Adherence, Risk factors

## Abstract

People living with HIV (PLWH) are particularly vulnerable to worsened outcomes of COVID-19. Therefore, the purpose of this work was to provide a scoping review of the literature to assess the risk factors for COVID-19 mortality among PLWH. Following the Preferred Reporting Items for Systematic Reviews and Meta-Analyses Extension for Scoping Reviews (PRISMA-ScR), searches were conducted in PubMed, Scopus, Global Health, and WHO Coronavirus Database. Articles were eligible for inclusion if they were in English, included PLWH who died after COVID-19 infection, and described risk factors for mortality. Results were descriptively synthesized and pooled thereafter. Study quality was assessed using the Joanna Brigg Institute’s critical appraisal tools. 20 studies were eligible for inclusion, with the pooled death rate being 11.7%. Age was a major risk factor, especially after 50 (23.2%) and after 70 (41.8%), and males had a death rate nearly double that of females. As total comorbidities increased, the death rate also greatly increased; among those with comorbidities, the highest fatality rates were those with cardiovascular disease (30.2%), chronic kidney disease (23.5%), obesity (22.4%), and diabetes (18.4%). Other risk factors for mortality among PLWH included having a Black racial background, being an injection drug user, being a smoker, and having a CD4 cell count below 200. There is a need to better study confounding factors, and to understand how vaccination influences mortality risk. Overall, the findings highlight a need to ensure that focus is placed on the varying demographics of PLWH amidst COVID-19 control efforts.

**Supplementary Information:**

The online version contains supplementary material available at 10.1007/s10461-022-03578-9.

## Introduction

The COVID-19 pandemic has immensely impacted the health status of diverse populations. As of December 23rd, 2021, there have been 276,436,619 confirmed cases of COVID-19 worldwide, and 5,374,744 deaths [1]. Though it has no restraint on who it affects, the trajectory of the pandemic’s effects and outcomes varies among populations. One such population that may be particularly vulnerable, due to their compromised immune statuses, is people living with HIV (PLWH).

As of 2020, there were 37,600,000 PLWH worldwide and 690,000 people who died due to HIV-related causes [2]. PLWH have a higher prevalence of comorbidities, higher mortality rate, are hospitalized at twice the rate of those without HIV and have a healthcare cost approximately four times those without HIV [3]. 73% of people living with HIV received antiretroviral therapies (ARTs) in 2020 [2]. As PLWH on ARTs live longer, many of them will have pre-existing chronic disease conditions which have been associated with severe COVID-19 co-infection [4]. Despite being on treatment, PLWH on ARTs consistently have a high rate of comorbidities including cardiovascular disease (CVD), sexually transmitted diseases, mental health conditions, neoplasms, diabetes, obesity, and chronic respiratory disease [4, 5].

The presence of comorbidities has consistently shown to be a risk factor for worsened outcomes among COVID-19 patients. Such comorbidities include hypertension, diabetes, CVD, and cerebrovascular disease [6]. However, prior systematic reviews focusing on whether HIV also increases likelihood of death by COVID-19 have provided conflicting findings [7–11]. Furthermore, these reviews have also offered limited insights regarding the risk factors for mortality [7–11].

In consideration of the uncertainty of previous findings, there is a need to better understand the relationship between COVID-19 among PLWH, and the overall risks for poor outcomes. While Mirzaei et al. (2021) [12] have completed a review on the clinical characteristics of patients coinfected with HIV and COVID-19, this was considerably earlier on in the pandemic and was limited to studies conducted before July 2020. Furthermore, that review was largely restricted to case reports and case series, and therefore had a limited total number of pooled patients [12]. This indicates that there is an urgent need to provide updated information in this area of research. The purpose of this study was hence to conduct a scoping review of the literature to provide an updated assessment of the risk factors for COVID-19 mortality among PLWH.

## Methods

This scoping review followed the Preferred Reporting Items for Systematic Review and Meta-Analyses Extension for Scoping Reviews (PRISMA-ScR) [13]. The steps involved for this project involved (1) developing the research question, (2) creating search terms in databases, (3) conducting searches, (4) selecting eligible studies, (5) charting of data and conducting quality assessments, (6) and describing and reporting of the findings. There was no registered study protocol for this review.

On July 20, 2021, searches were conducted in four different databases: PubMed, Scopus, WHO Coronavirus Database, and Global Health. As our population of interest was PLWH who died of COVID-19, searches included relevant terms on HIV, COVID-19, and mortality. No restrictions were placed based on date or language in these searches. Full search terms, with the respective database, are listed in Supplementary Table 1.

Original research studies eligible for inclusion were required to have a minimum of five PLWH who died while infected with COVID-19 and provided stratified data with characteristics of the patients who died. Studies were also required to be in English. To keep the inclusion criteria as broad as possible, peer-reviewed articles and pre-print papers were eligible, and there were no restrictions placed on study type, or date of publication.

After completion of the initial searches, two researchers (KV and PG) independently screened articles. Duplicates were first removed, and articles were thereafter screened by title and abstract. The remaining articles underwent full-text analysis and were excluded if they did not meet the criteria for inclusion. Discrepancies of selected articles among the two researchers were evaluated until consensus was reached about the final articles for inclusion.

Study characteristics, as well as patient data, were next extracted from the final articles. The characteristics of the studies considered were city and country of the study, source of data, study design, and proportion of deaths among PLWH infected with COVID-19. Patient data that was extracted included patient total, sex, age, race, comorbidities, HIV viral load, CD4 cell count (per mm^3^), as well as additional features relevant to patient outcomes that were discussed in the study. The additional features included (but were not limited to) the following: a history of an AIDS diagnosis, men who have sex with men (MSM), injection drug use (IDU), smoking status, and having had the influenza vaccination. Thereafter, a pooled analysis was conducted to determine the overall and stratified case-fatality rate. Data was synthesized and analysed descriptively, with the use of MS excel sheets.

All included studies were assessed for methodological quality using the Joanna Briggs Institute’s (JBI) critical appraisal tools [14]. As has been conducted in other reviews [15, 16], the tools were altered to provide a numeric score, with cohort studies on an eleven-item scale, case reports on an eight-item scale, case series on a ten-item scale, and cross-sectional studies on an eight-item scale. These numeric scores were used to compare differences in methodological quality across studies.

## Results

**Fig. 1:**
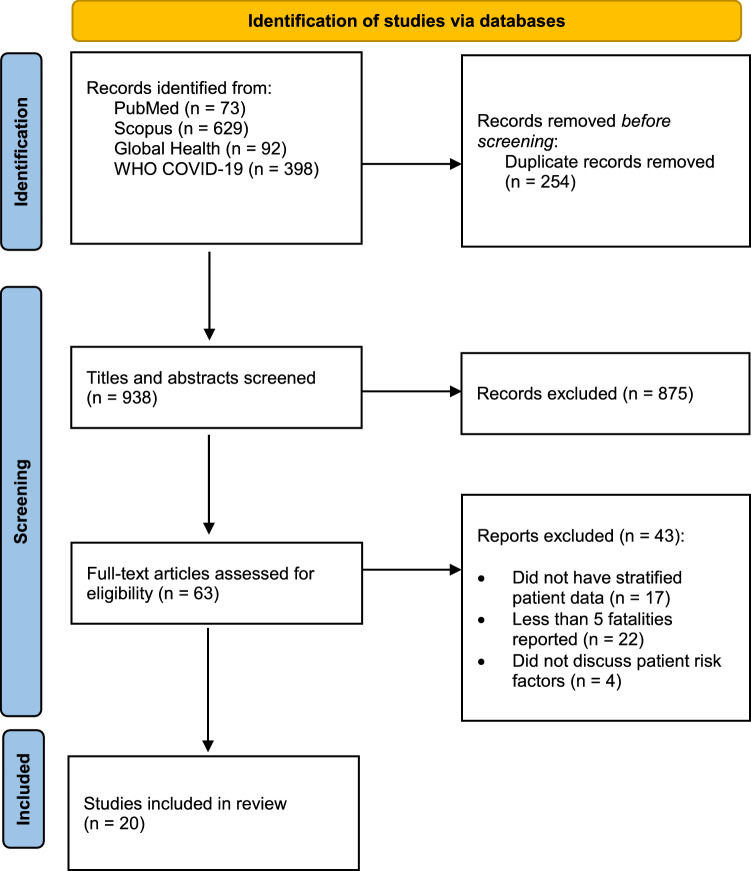
Process of searching and selecting articles included in the scoping review based on the PRISMA 2020 flow diagram [13]

Searches from all databases produced a total of 1192 articles. 938 articles remained after removal of duplicates, and after screening by title and abstract, 63 articles remained. A total of 20 articles [17–36] met the inclusion criteria and hence were included in our analysis; 43 articles were removed, with reason. Figure 1 shows the entire screening process for this review.

Characteristics of the included studies are listed in Table 1. Studies were conducted in seven different countries: United States of America (USA) (n = 10) [19, 22, 25, 27–29, 32–35], South Africa (SA) (n = 4) [18, 26, 30, 36], United Kingdom (UK) (n = 2) [17, 24], Brazil (n = 1) [31], Chile (n = 1) [20], Zambia (n = 1) [21], and Spain (n = 1) [23]. 18 of the studies had a cohort design [17–29, 31–33, 35, 36], whereas there was one case series [34], and one cross-sectional analysis [30].

**Table 1:** Characteristics of included studies

Study	Country	City	Source of data	Study design	Deaths/total cases of COVID-19 among PLWH (% of total)	Study ranking
Bhaskaran et al. [17]	UK	Across the UK	OpenSAFELY, a data platform to understand COVID-19; electronic data from primary care practices with The Phoenix Partnership (TPP) SystemOne Software	Retrospective cohort study	25 deaths; total cases not specified	9/11
Boulle et al. [18]	SA	Across Western Cape Province	Data from health facilities of the public sector in Western Cape	Retrospective cohort study	115/3978 (2.9)	10/11
Braunstein et al. [19]	USA	New York City	COVID-19 case and death data from the New York City Health Department, against the New York City HIV surveillance registry	Retrospective cohort study	312/2410 (12.9)	9/11
Ceballos et al. [20]	Chile	Across the nation	COVID-19 data from 23 hospitals across Chile	Prospective cohort study	5/36 (13.9)	7/11
Chanda et al. [21]	Zambia	Lusaka, Ndola, Kabwe, Livingstone	Five Zambia Ministry of Health specialized COVID-19 treatment centers	Retrospective cohort study	17/122 (13.9)	6/11
Dandachi et al. [22]	United States	Across the nation	A multicenter registry containing chart data from Infectious Disease departments and HIV clinics	Retrospective cohort study	27/164 (16.5)	7/11
del Amo et al. [23]	Spain	Madrid	HIV clinics of hospitals, 2019 National HIV Hospital Survey, and COVID-19 Health information system	Retrospective cohort study	20/236 (8.5)	8/11
Geretti et al. [24]	UK: England, Scotland, and Wales	Across England, Scotland, and Wales	Data from participating hospitals in these regions	Prospective cohort study	30/122 (24.6)	8/11
Ho et al. [25]	USA	New York City	Electronic medical records from five emergency departments	Retrospective cohort study	19/93 (20.4)	10/11
Jassat et al. [26]	SA	Across the nation	A national surveillance system for COVID-19 hospitalizations by the National Institute for Communicable Diseases	Retrospective cohort study	644/3077 (20.9)	8/11
Karmen-Tuohy et al. [27]	USA	New York City	Electronic medical data from New York University Langone Health	Retrospective cohort study	6/21 (28.6)	7/11
Marcello et al. [28]	USA	New York City	Medical records for patients who tested positive for COVID-19 at any NYC H+H location	Prospective cohort study	20/94 (21.3)	9/11
Miyashita and Kuno [29]	USA	New York City	Electronic medical records of Mount Sinai Health System with data	Retrospective cohort study	23/161 (14.3)	7/11
Pillay-van Wyk et al. [30]	SA	Across the nation	COVID-19 death reports from the National Department of Health	Cross-sectional analysis	342/2457 (13.9)	5/8
Rocha et al. [31]	Brazil	San Paulo	COVID-19 cases reported to the Sao Paulo State surveillance system and Brazilian Ministry of Health surveillance, as well as the national HIV surveillance	Retrospective cohort study	83/255 (32.5)	9/11
Shalev et al. [32]	USA	New York City	Medical records from a large tertiary medical care center	Retrospective cohort study	8/31 (25.8)	6/11
Sigel et al. [33]	USA	New York City	Electronic health data from five hospitals in the Mount Sinai Health System	Retrospective cohort study	18/88 (20.5)	9/11
Suwanwongse and Shabarek [34]	USA	New York City	Health data from a single hospital in South Bronx, New York City	Case Series	7/9 (77.8)	6/10
Tesoriero et al. [35]	USA	Across New York State	New York State HIV Surveillance registry, New York State Electronic Clinical Laboratory Reporting System, and the state Health Information Network	Retrospective cohort study	207/2988 (6.9)	8/11
Venturas et al. [36]	SA	Johannesburg	Medical records from the Charlotte Maxeke Johannesburg Academic Hospital	Retrospective cohort study	16/108 (14.8)	8/11
Pooled total deaths: 1944 Pooled total cases*^1^: 16,450 Pooled mortality rate*^2^: 1919/16,450 = 11.7%						

Created by the authors

*^1^: Excluding studies where this was not reported

*^2^: Excluded death totals for studies where total cases was not reported

**Fig. 2:**
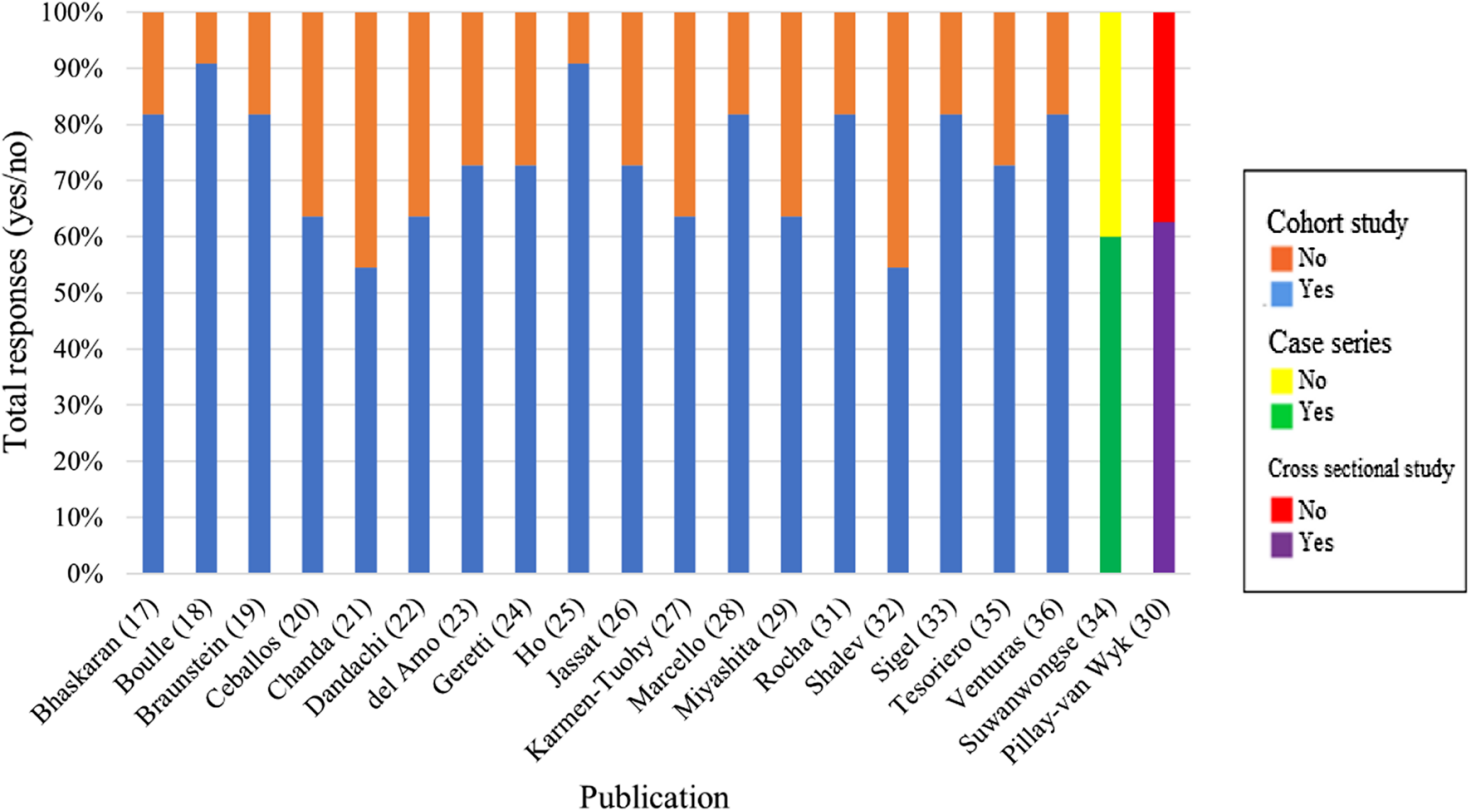
Quality assessment scores for included publications reported as “yes” or “no” for achieving quality metrics per the Joanna Briggs Institute’s critical appraisal tools. *Created by the authors*

Quality assessment scores for the cohort studies ranged from 6/11 to 10/11 (mean = 8.1), 5/8 for the cross-sectional study, and 6/10 for the case series. Figure 2 depicts the scores (% yes/no) for the quality assessments. The most frequent study design limitations involved issues with describing factors contributing to loss to follow-up, a lack of utilization of strategies to address incomplete follow-up, limited identification of confounding factors, and a lack of strategies to address confounding factors if they had been identified. Full quality assessment checklists are listed in Supplementary Tables 2–4.

Case-fatality rates among studies ranged from 2.9 [18] to 32.5% [31]. A single study did not include total cases of COVID-19 among PLWH [17]. The findings of the pooled analysis are listed in Table 2. The pooled case-fatality rate for cases of COVID-19 among PLWH was 11.7% (1919 deaths among 16,450 cases). Patient characteristics for all of those who died in each study are listed in Supplementary Table 5.

**Table 2:** Pooled analysis of case-fatality rate by risk factor

Risk factor	Deaths/cases (%)
Total	1919/16,450 (11.7)
Race	
Black	337/2604 (12.9)
Hispanic/Latino	239/2321 (10.3)
White	74/738 (10.0)
Other	29/348 (8.3)
Sex	
Male	537/5300 (10.1)
Female	265/4878 (5.4)
Age	
70+	82/196 (41.8)
60+	398/2015 (19.5)
50+	467/2015 (23.2)
40+	614/5540 (11.1)
< 40	34/2830 (1.2)
50–59	60/594 (10.1)
40–49	34/1244 (2.7)
Viral load	
Virally suppressed	544/3844 (14.2)
Virally unsuppressed	97/659 (14.7)
CD4 count (per mm^3^)	
200+	453/1974 (22.9)
< 200	251/733 (34.2)
Comorbidities	
1 + comorbidities	424/1877 (22.3)
2 + comorbidities	43/90 (47.8)
3 + comorbidities	19/31 (61.3)
Hypertension	62/784 (7.9)
Diabetes	79/430 (18.4)
Cardiovascular disease (other than hypertension)	13/43 (30.2)
Obesity	13/58 (22.4)
Chronic kidney disease (CKD)	32/136 (23.5)
Chronic obstructive pulmonary disease (COPD)	17/254 (6.7)
Cancer	3/23 (13.0)
Neuropsychiatric disease	3/12 (25.0)
Previous organ transplant	3/4 (75.0)
Hyperlipidemia	3/4 (75.0)
Chronic liver disease	1/3 (33.3)
Past/current tuberculosis	59/1102 (5.4)
Hepatitis C	2/3 (66.6)
Syphilis	0/1 (0.0)
Bacterial superinfection	3/3 (100.0)
Influenza vaccination received	12/44 (27.3)
History of AIDS diagnosis	
Yes	249/742 (33.6)
No	63/269 (23.4)
Current/past smoker	15/54 (27.8)
Men who have sex with men (MSM)	133/1875 (7.1)
Injection drug user (IDU)	126/803 (15.7)
MSM & IDU	14/20 (70.0)

Created by the authors

Amongst racial groups, it was found that Black individuals had the highest fatality rate (12.9%), followed by Hispanic/Latino individuals (10.3%), then White individuals (10.0%), with people of other races having the lowest rate (8.3%). Total COVID-19 case rates were also highest amongst Blacks, followed by Hispanics/Latinos.

Males were found to have a mortality rate nearly double that of females (10.1% and 5.4% respectively). In terms of age, there was a consistent trend showing that an increase in age corresponds to a markedly higher risk of mortality. These differences were largest amongst those 70 years and above, who had a case-fatality rate of 41.8%, compared to those under 40 years of age, who had a fatality rate of 1.2%. There was also found to be a near five times increase in death rates for those above the age of 50, with those of age 40–49 having a rate of 2.7%, and those 50–59 having a rate of 10.1%.

In the pooled analysis, death rates among those with and without viral suppression were relatively similar, at 14.2% amongst those virally suppressed, and 14.7% for those who were virally unsuppressed. A lower CD4 cell count did have an impact on fatality rates; those with a CD4 count under 200 had a death rate of 34.2%, compared to 22.9% for those with a CD4 count above 200. A single study, which compared fatality rates amongst patients, showed that those with a viral load above 1000 copies/mL or a CD4 count below 200 had an adjusted hazards ratio of 3.80 (95% CI: 2.07, 6.95) [18].

The presence of comorbidities also led to a higher death rate among patients. Those with one or more comorbidities had a fatality rate of 22.3%, and this increased to 47.8% for patients with two or more, and 61.3% for those with three or more. CVD (other than hypertension) patients had an elevated case-fatality rate of 30.2%, as well as diabetes patients (18.4%), obese patients (22.4%), and CKD patients (23.5%). Patients with hypertension had lower death rates (7.9%), along with patients with chronic obstructive pulmonary disease (COPD) (6.7%) and current or past tuberculosis (5.4%).

There were varying trends for the additional factors. Patients with a history of an AIDS diagnosis had a fatality rate of 33.6%, though this factor was only evaluated in a single study [19]; in that study, patients without an AIDS diagnosis also had a death rate above the pooled fatality rate (23.4%). Patients with the following factors also had elevated death rates: current/past smoker (27.8%), being vaccinated for influenza (27.3%), bacterial superinfection (100.0%), IDU (15.7%), IDU amongst MSM (70.0%). MSM, as a whole, had lower death rates (7.1%).

## Discussion

The findings of this scoping review provide further evidence indicating that PLWH are at a high risk of COVID-19 mortality, with the case-fatality rate among PLWH patients included in this review being 11.7%. Based on these high mortality rates, it is evident that extra support and services are needed during the pandemic in order improve outcomes for this population. One way this can happen is with the expansion of telemedicine services for this demographic. Considering that telemedicine services have been shown in previous studies to be effective in supporting PLWH both prior to [37, 38], and during [39, 40], the COVID-19 pandemic, expansion of these services may be capable of improving patient outcomes. Additionally, increasing access to counselling services, pharmacy services, and social services have been shown to be beneficial to PLWH during the COVID-19 pandemic [41]. Scaling up of these services is hence recommended, alongside studies to determine their overall effectiveness in lowering mortality rates.

Our review has shown that a substantial proportion of PLWH were either virally unsuppressed or had a low CD4 cell count; those with a lower CD4 count had a particularly high risk of death. A possible explanation for this is an interruption of adherence to ARTs due to social distancing measures during the COVID-19 pandemic, as this is a problem that has been described in numerous different contexts [6, 42]. Alongside a need for more research on this issue, this indicates that considerations should be made for scaling up of HIV treatment adherence programs alongside increased pandemic control efforts. One such program, which has previously been shown to be effective in meeting emergency needs for PLWH in a variety of contexts during the COVID-19 pandemic, involves the provision of ARTs via home delivery [43]. Programs such as this could possibly be expanded to help to ensure that PLWH also receive other services and resources, such as food, masks, and other essential items.

When considering race, it is evident that PLWH who are Black are at a particularly elevated risk of contracting, and dying from, COVID-19 compared to those of other racial groups. These findings have important implications, and further highlight the consequences of racial inequities during the COVID-19 pandemic. Our work hence shows that there is a need to create more programs that are specifically directed towards helping Black individuals amidst the pandemic, particularly across the United States. Therefore, targeted vaccination campaigns, along with resource allocation programs, have the potential to have a sizeable impact. Furthermore, prior research has indicated that medical mistrust regarding COVID-19 among HIV-positive Black individuals may be a contributor to health inequities [44]. Addressing this mistrust with community engagement may also be pivotal in improving COVID-19 outcomes among HIV-positive Black individuals [44].

There is a striking difference in death rates among people of different sexes, and for people of different age groups. Males were nearly twice as likely to die from COVID-19 than females, and PLWH above the age of 70 had a death rate nearly 35 times higher than those under 40. It is worthwhile denoting that comparable trends have also been seen among COVID-19 patients not living with HIV [45]. Studying how gender may influence mortality rates and ensuring that older PLWH with COVID-19 have increased access to care will therefore be important.

Based on the findings, it is unclear if MSM are at a higher risk of death. The findings do however show that IDU are at a higher risk of death, and risk of fatality is especially high amongst IDU who are also MSM. Previous research has provided an indication that, during the COVID-19 pandemic, IDU have had lower ART adherence rates and increased rates of illicit substance use overall, and this may be attributed to increased social-distancing measures [46]. There is hence a clear need to support IDU during the pandemic by creating and scaling-up programs that can offer harm-reduction, syringe exchange, and easier access to ARTs [46–50]. It has also been proposed that mobile-health interventions may serve as an effective way to support IDU during pandemics [46], but the evidence for this is limited. Based on this, it is also recommended that future research assess and evaluate the type of interventions that can most optimally lower mortality rates of IDU living with HIV and COVID-19.

The presence of comorbidities was shown to correspond to high death rates, with those living with multiple comorbidities having especially high rates of death. CVD, obesity, CKD, and diabetes are all conditions which appear to elevate death rates, along with smoking. However, an opposite pattern was observed for those with hypertension, COPD, and tuberculosis. Furthermore, there was limited patient data for those with cancer, neuropsychiatric disease, organ transplants, hyperlipidemia, and bacterial superinfection. This emphasizes a need to better understand the risk due to specific comorbidities for this demographic. Similarly, it would be beneficial to better understand the effects of influenza vaccinations, and whether they may be beneficial, or even detrimental for this group.

Notably, of the studies included in this review, there were no case–control studies included. This hence restricted the capability to compare outcomes for COVID-19 patients who did, and did not, have HIV. Additionally, while there were a small number of high-quality studies, many of the included studies were either of moderate or low quality and did not appropriately account for confounding factors. It is hence recommended that more high-quality studies, with controls, be conducted to better understand the factors that place PLWH at risk of COVID-19 mortality.

There are several important limitations to consider. First, none of the studies provided data on the effects of COVID-19 vaccination, and the extent to which this impacts risk. Secondly, as half of the studies are from USA, it is not entirely clear how much findings from these settings can be generalized to other nations. Furthermore, the findings do not provide a clear indication as to whether having an elevated viral load influences overall fatality rates; considering that many of the social distancing measures during the COVID-19 pandemic have previously shown to impact medication adherence [6, 42], there is a clear need to better understand the implications of this on COVID-19 outcomes. It is hence recommended that future studies be conducted to determine if ART adherence rates have decreased, and the reasons for this. This will be crucial, as emphasized by the fact that those with a lower CD4 cell count had a particularly high risk of death.

## Conclusions

PLWH are at a high risk for death by COVID-19. Risk factors for death were found to be having a Black racial background, living with comorbidities, being an IDU, being older, having a low CD4 cell count, and being male. Targeted interventions and pandemic control efforts towards individuals with these risk factors will be imperative to effectively save lives of PLWH during the COVID-19 pandemic.

## Supplementary Information

Below is the link to the electronic supplementary material.

Supplementary file1 (DOCX 38 kb)
