# Supplementary material for: Risk Factors for COVID-19 Mortality Among People Living with HIV: A Scoping Review
Source: AIDS Behav. 2022 Jan 13;26(7):2256–65. doi: 10.1007/s10461-022-03578-9 (PMC8756751; doi:10.1007/s10461-022-03578-9)
Supplement: Supplementary file 1 — Supplementary file1 (DOCX 38 kb) [file 10461_2022_3578_MOESM1_ESM.docx]

**Supplementary Table 1: Databases & search terms**

| **PubMed** | ((severe acute respiratory syndrome coronavirus 2 [Title/Abstract] OR 2019 Novel coronavirus [Title/Abstract] OR 2019-nCoV infection [Title/Abstract] OR 2019 novel coronavirus infection [Title/Abstract] OR coronavirus disease 2019 virus [Title/Abstract] OR wuhan coronavirus [Title/Abstract] OR coronavirus [Title/Abstract] OR Novel coronavirus [Title/Abstract] OR coronavirus disease [Title/Abstract] OR 2019-ncov [Title/Abstract] OR COVID-19 [Mesh] OR SARS-CoV-2 [Mesh])) AND ("HIV Infections"[Mesh] OR "HIV"[Mesh] OR "Acquired Immunodeficiency Syndrome"[Mesh] OR HIV[tiab] OR HIV-1[tiab] OR HIV-2[tiab] OR human immunodeficiency virus[tiab] OR human immunedeficiency virus[tiab] OR human immuno-deficiency virus[tiab] OR human immune-deficiency virus[tiab] OR (human immun*[tiab] AND deficiency virus[tiab]) OR acquired immunodeficiency syndrome[tiab] OR acquired immuno-deficiency syndrome[tiab] OR acquired immune-deficiency syndrome[tiab] OR (acquired immun*[tiab] AND deficiency syndrome[tiab])) AND ("Death"[Mesh] OR "Mortality"[Mesh] OR "died"[Title/Abstract]) |
| --- | --- |
| **Scopus** | (TITLE-ABS-KEY ( {HIV Infections} ) OR TITLE-ABS-KEY ( {HIV} ) OR TITLE-ABS-KEY ( { Acquired Immunodeficiency Syndrome} ) OR TITLE-ABS-KEY ( { HIV-1} ) OR TITLE-ABS-KEY ( { HIV-2} ) OR TITLE-ABS-KEY ( { human immunodeficiency virus } ) OR TITLE-ABS-KEY ( { human immunedeficiency virus} ) OR TITLE-ABS-KEY ( { acquired immuno-deficiency syndrome} ) ) AND ( TITLE-ABS-KEY ( {severe acute respiratory syndrome coronavirus 2} ) OR TITLE-ABS-KEY ( {Novel coronavirus} ) OR TITLE-ABS-KEY ( {2019-nCoV infection} ) OR TITLE-ABS-KEY ( {2019 novel coronavirus infection} ) OR TITLE-ABS-KEY ( {coronavirus disease 2019 virus} ) OR TITLE-ABS-KEY ( {wuhan coronavirus} ) OR TITLE-ABS-KEY ( {coronavirus} ) OR TITLE-ABS-KEY ( {Novel coronavirus} ) OR TITLE-ABS-KEY ( {coronavirus disease} ) OR TITLE-ABS-KEY ( {covid 2019} ) OR TITLE-ABS-KEY ( {covid-19} ) OR TITLE-ABS-KEY ( {sars-cov2} ) OR TITLE-ABS-KEY ( {2019-ncov} ) ) AND ( TITLE-ABS-KEY ( {Death} ) OR TITLE-ABS-KEY ( {Died} ) OR TITLE-ABS-KEY ( { Mortality} ) ) AND ( LIMIT-TO ( PUBYEAR , 2021 ) OR LIMIT-TO ( PUBYEAR , 2020 ) OR LIMIT-TO ( PUBYEAR , 2019 )) |
| **WHO Coronavirus Database** | "HIV" AND ("mortality" OR "death" OR "died") AND ("COVID-19" OR "coronavirus") |
| **Global Health** | (“HIV Infections” OR “HIV Acquired Immunodeficiency Syndrome” OR “HIV-1” OR “HIV-2 OR human immunodeficiency virus” OR “human immunedeficiency virus” OR “acquired immuno-deficiency syndrome” ) AND (“severe acute respiratory syndrome coronavirus 2” OR “Novel coronavirus” OR “2019-nCoV infection” OR”2019 novel coronavirus infection” OR “coronavirus disease 2019 virus” OR “wuhan coronavirus” OR ”coronavirus” OR “Novel coronavirus” OR “coronavirus disease” OR “covid 2019” OR “covid-19” OR”sars-cov2” OR “2019-ncov”) AND (“Death” OR “Died” OR Mortality”) |

**Supplementary Table 2. Joanna Briggs Quality Assessment for analytical cross-sectional studies included in the review**

| First Author (Ref) | 1 | 2 | 3 | 4 | 5 | 6 | 7 | 8 | Total score |
| --- | --- | --- | --- | --- | --- | --- | --- | --- | --- |
| **Pillay-van Wyk (30)** | Yes | No | Yes | Yes | No | No | Yes | Yes | 5/8 |

1. Were the criteria for inclusion in the sample clearly defined?
2. Were the study subjects and the setting described in detail?
3. Was the exposure measured in a valid and reliable way?
4. Were objective, standard criteria used for measurement of the condition?
5. Were confounding factors identified?
6. Were strategies to deal with confounding factors stated?
7. Were the outcomes measured in a valid and reliable way?
8. Was appropriate statistical analysis used?

**Supplementary Table 3. Joanna Briggs Quality Assessment for case-series included in the review**

| First Author (Ref) | 1 | 2 | 3 | 4 | 5 | 6 | 7 | 8 | 9 | 10 | Total Score |
| --- | --- | --- | --- | --- | --- | --- | --- | --- | --- | --- | --- |
| **Suwanwongse (34)** | Yes | Yes | Yes | No | Yes | No | Yes | Yes | No | No | 6/10 |

1. Were there clear criteria for inclusion in the case series?
2. Was the condition measured in a standard, reliable way for all participants included in the case series?
3. Were valid methods used for identification of the condition for all participants included in the case series?
4. Did the case series have consecutive inclusion of participants?
5. Did the case series have complete inclusion of participants?
6. Was there clear reporting of the demographics of the participants in the study?
7. Was there clear reporting of clinical information of the participants?
8. Were the outcomes or follow up results of cases clearly reported?
9. Was there clear reporting of the presenting site(s)/clinic(s) demographic information?
10. Was statistical analysis appropriate?

**Supplementary Table 4. Joanna Briggs Quality Assessment for cohort studies included in the review**

| First Author (Ref) | 1 | 2 | 3 | 4 | 5 | 6 | 7 | 8 | 9 | 10 | 11 | Total score |  |
| --- | --- | --- | --- | --- | --- | --- | --- | --- | --- | --- | --- | --- | --- |
| **Bhaskaran (17)** | Yes | Yes | No | Yes | Yes | Yes | Yes | Yes | Yes | No | Yes | 9/11 |  |
| **Boulle (18)** | Yes | Yes | Yes | Yes | Yes | Yes | Yes | Yes | Yes | No | Yes | 10/11 |  |
| **Braunstein (19)** | Yes | Yes | Yes | Yes | Yes | Yes | Yes | Yes | No | No | Yes | 9/11 |  |
| **Ceballos (20)** | Yes | Yes | Yes | No | No | Yes | Yes | Yes | Yes | No | No | 7/11 |  |
| **Chanda (21)** | Yes | Yes | Yes | Yes | Yes | No | No | No | No | No | Yes | 6/11 |  |
| **Dandachi (22)** | Yes | Yes | Yes | No | No | Yes | Yes | No | Yes | No | Yes | 7/11 |  |
| **del Amo (23)** | Yes | Yes | Yes | No | No | Yes | Yes | Yes | Yes | No | Yes | 8/11 |  |
| **Geretti (24)** | Yes | Yes | No | No | No | Yes | Yes | Yes | Yes | Yes | Yes | 8/11 |  |
| **Ho (25)** | Yes | Yes | Yes | Yes | No | Yes | Yes | Yes | Yes | Yes | Yes | 10/11 |  |
| **Jassat (26)** | Yes | Yes | Yes | Yes | Yes | Yes | Yes | No | No | No | Yes | 8/11 |  |
| **Karmen-Tuohy (27)** | Yes | Yes | Yes | No | No | Yes | Yes | No | Yes | No | Yes | 7/11 |  |
| **Marcello (28)** | Yes | Yes | Yes | Yes | Yes | Yes | Yes | Yes | No | No | Yes | 9/11 |  |
| **Miyashita (29)** | Yes | Yes | Yes | No | No | Yes | Yes | Yes | No | No | Yes | 7/11 |  |
| **Rocha (31)** | Yes | Yes | Yes | Yes | Yes | Yes | Yes | Yes | No | No | Yes | 9/11 |  |
| **Shalev (32)** | Yes | Yes | Yes | No | No | Yes | Yes | Yes | No | No | No | 6/11 |  |
| **Sigel (33)** | Yes | Yes | Yes | Yes | Yes | Yes | Yes | No | Yes | No | Yes | 9/11 |  |
| **Tesoriero (35)** | Yes | Yes | Yes | Yes | Yes | Yes | Yes | No | No | No | Yes | 8/11 |  |
| **Venturas (36)** | Yes | Yes | Yes | Yes | Yes | Yes | Yes | No | No | No | Yes | 8/11 |  |

1. Were the two groups similar and recruited from the same population?
2. Were the exposures measured similarly to assign people to both exposed and unexposed groups?
3. Was the exposure measured in a valid and reliable way?
4. Were confounding factors identified?
5. Were strategies to deal with confounding factors stated?
6. Were the groups/participants free of the outcome at the start of the study (or at the moment of exposure)?
7. Were the outcomes measured in a valid and reliable way?
8. Was the follow up time reported and sufficient to be long enough for outcomes to occur?
9. Was follow up complete, and if not, were the reasons to loss to follow up described and explored?
10. Were strategies to address incomplete follow up utilized?
11. Was appropriate statistical analysis used?

**Supplementary Table 5: Mortality data by study for PLWH infected with COVID-19**

| **Study** | **Total deceased** | **Sex (M / F)** | **Age** | **Race** | **Comorbidities** | **HIV viral load** | **CD4 cell count** | **Additional features** |
| --- | --- | --- | --- | --- | --- | --- | --- | --- |
| *Bhaskaran et al (17)* | 25 | 18 / 7 | 60+: 15  60 or less: 10 | Black: 11  Other race, or unknown; 14 | Hypertension: 15  Diabetes: 14  Reduced kidney function: 9  Obese: 10  At least one comorbidity: 23 | N/A | N/A | N/A |
| *Boulle et al (18)* | 115 | 53 / 62 | 70+: 9  60-69: 21  50-59: 40  40-49: 28  20-39: 17 | N/A | Diabetes: 58  Hypertension: 48  Chronic kidney disease: 21  Chronic pulmonary disease / asthma: 10  Previous tuberculosis: 42  Current tuberculosis: 16 | VL > 1000 copies/ml or CD4 <200/ μl (last 18 months): aHR = 3.80 (95% CI: 2.07, 6.95)  VL < 1000 copies/ml (last 15 months) & ART script (last 6 months): aHR = 1.75 (95% CI: 1.34, 2.29)  No VL (last 15 months) or CD4 >200cells/ μl or unknown (last 18 months): aHR = 1.54 (95% CI: 1.01, 2.33) | VL > 1000 copies/ml or CD4 <200/ μl (last 18 months): aHR = 3.80 (95% CI: 2.07, 6.95)  No VL (last 15 months) or CD4 >200cells/ μl or unknown (last 18 months): aHR = 1.54 (95% CI: 1.01, 2.33) | ART duration:  <1 year: (ref)  1-2 years: aHR = 0.78 (95% CI: 0.25, 2.47)  2+ years: aHR = 0.47 (95% CI: 0.20, 1.12) |
| *Braunstein et al (19)* | 312 | 224 / 88 | 75+: 56  65-74: 88  45-64: 150  18-44: 18 | Black: 153  Latino/Hispanic: 124  White: 31  Asian/Pacific Islander: 4 | 1+ comorbidity: 292 | 100,000+ cc/mL: 5  10,000-99,999 cc/mL: 4  1,500-9,999 cc/ml: 2  20-1499 cc/mL: 10  <200 cc/mL: 281  Not reported: 10  HIV virally suppressed at last viral load: 281 | 500+ cells/μl: 81  350-499 cells/μl: 51  200-349 cells/μl: 73  <200 cells/μl: 99  Not reported: 8 | Men who have sex with men (MSM): 79  Injection drug-use history (IDU): 71  MSM-IDU: 7  History of AIDS diagnosis: 249  No history of AIDS diagnosis: 63  Residing in area with very high poverty (30% below federal poverty line): 128  Residing in area with high poverty (20 - <30% below federal poverty line): 73  Residing in area with medium poverty (10 - <20% below federal poverty line): 94  Residing in area with low poverty (<10% below federal poverty line): 17  Diagnosed 2010-2020: 28  Diagnosed 2000-2009: 114  Diagnosed 1991-1999: 130  Diagnosed: 1990 or before: 40 |
| *Ceballos et al. (20)* | 5 | 4 / 1 | Mean age: 57 | N/A | Diabetes: 2  Hypertension: 3  Obesity: 1  Cancer: 1  Cardiovascular disease: 3  Chronic kidney disease: 2  At least one comorbidity: 4 | HIV viral load < 50 copies RNA/mL before admission: 4 | Mean baseline CD4 count before admission, cells/mm^3^: 543  Mean in-hospital CD4 count, cells/mm^3^: 344 | On ART: 4  Median time since HIV diagnosis, years: 16  Length of hospital stay, days: 19  Oxygen requirement: 5 |
| *Chanda et al. (21)* | 17 (adjusted hazard ratios, with comparison of patients with controlled HIV) | Male sex: aHR = 1.71 (95% CI: 1.07 – 2.76) | Age 60+: aHR = 2.09 (95% CI: 1.32 – 3.29) | N/A | Two or more underlying medical conditions: aHR = 1.78 (95% CI: 1.11 – 2.83) | N/A | N/A | Severe HIV disease (if they had one or more of the following: severely anemic, CD4 < 200 cells/μl, active TB, or underweight (BMI < 18.5 kg/m^2^): aHR = 3.27 (95% CI: 1.21 – 8.79) |
| *Dandachi et al (22)* | 27 | N/A | 60+: 18  40-60: 8  <40: 1 | N/A | N/A | N/A | N/A | N/A |
| *del Amo et al (23)* | 20 | 16 / 4 | 70-79: 7  60-69: 4  50-59: 7  40-49: 2 | N/A | N/A | N/A | N/A | NRTI – TAF/FTC: 10  NRTI – ABC/3TC: 8  NNRTI: 5  Protease inhibitor: 5  Integrase inhibitor: 9 |
| *Geretti et al (24)* | 30 | N/A | 70+: 7  60-69: 6  50-59: 10  40-49: 4  40 or less: 1 |  | Chronic pulmonary disease: 1  Diabetes: 5  Obesity: 8 | N/A | N/A | On ARTs: 25  Presenting with cough: 26  Presenting with diarrhea: 8  Tachycardia: 16  Tachypnea: 18  Oxygen therapy provided: 22  Critical care admission: 20  Noninvasive ventilation provided: 12  Invasive ventilation provided: 13 |
| *Ho et al (25)* | 19 | 13 / 6 | Mean age: 62 | White: 7  Black: 2  Hispanic: 6  Unspecified race: 10 | Obesity: 3 | Plasma HIV RNA suppressed: 9 | Mean Nadir CD4 T cell count, cells/μl: 341  Mean CD4 T cell count preceding COVID-19 diagnosis, cells/μl: 686  Mean Presenting CD4 T cell count during hospitalization, cells/μl: 173 | Mean duration of HIV infection (years): 22  Current smoker: 2  Admitted to ICU: 13  O2 ventilator: 11  Mean duration of hospitalization (days): 12 |
| *Jassat et al (26)* | 644 | N/A | N/A | N/A | 3+ comorbidities: 19  2 comorbidities: 4  1 comorbidity: 66  1+ comorbidities: 128  No comorbidity: 46  Not reported: 470 | Viral load 1000+: 39  Viral load less than 1000: 210  Not reported: 395 | CD4 200+: 179  CD4 <200: 128  Not reported: 306 | On ART: 290  Not on ART: 1  ART not reported: 353 |
| *Karmen-Tuohy et al (27)* | 6 | N/A | N/A | N/A | N/A | N/A | N/A | Bacterial superinfection: 3 |
| *Marcello et al (28)* | 20 | N/A | N/A | Hispanic: 9  Black: 8  White: 1  Other racial status: 2 | N/A | N/A | N/A | N/A |
| *Miyashita & Kuno (29)* | 23 | N/A | 66+: 8  51-65: 12  50 or less: 3 | N/A | N/A | N/A | N/A | N/A |
| *Pillay-van Wyk (30)* | 342 | 162 / 180 | 70+: 22  50-69: 162  <50: 158  Median age (years): 51 | N/A | Diabetes and hypertension: 35  Only diabetes: 3 | N/A | N/A | In Western Cape province: 259  In Eastern Cape province: 29  In Gauteng province: 37  Other provinces: 17 |
| *Rocha et al (31)* | 83 | 60 / 23 | 60+: 34  40-59: 38  20-39: 11 | Black / mixed: 71  White / Caucasian / Asian: 10 | Median number of comorbidities: 1 | Viral load <50copies/mL (undetectable): 51 | CD4 350+ per mm^3^: 48  CD4 200-349 per mm^3^: 9  CD4 <200 per mm^3^: 12 | ART: 64  Received influenza vaccine in 2020: 12  Time between symptom onset and death: 18 days  Education <8 years: 69  Education 8+ years: 12 |
| *Shalev et al. (32)* | 8 | N/A | 65+: 4  50-65: 4 | N/A | N/A | N/A | N/A | ART: 7  Tenofovir: 7 |
| *Sigel et al (33)* | 18 | 13 / 5 | Median age (years): 62 | White: 5  Black: 3  Hispanic: 8  Other race: 2 | Diabetes: 4  Hypertension: 6  Obesity: 1  Chronic obstructive pulmonary disease: 2  Cirrhosis: 1  Chronic kidney disease: 6  Organ transplant: 3  Cancer: 1 | HIV RNA level >50, copies/μl, at admission: 1  HIV RNA level <50, copies/μl, at admission: 12  HIV RNA level >50, copies/μl, prior to admission: 1  HIV RNA level <50, copies/μl, prior to admission: 10 | CD4 cell count, <50 cells/mm^3^, at admission: 1  CD4 cell count, 50-200 cells/mm^3^, at admission: 6  CD4 cell count, 201-500 cells/mm^3^, at admission: 6  CD4 cell count, 500+ cells/mm^3^, at admission: 0  CD4 cell count, <50 cells/mm^3^, prior to admission: 0  CD4 cell count, 50-200 cells/mm^3^, prior to admission: 3  CD4 cell count, 201-500 cells/mm^3^, prior to admission: 2  CD4 cell count, 500+ cells/mm^3^, prior to admission: 3 | Current or former smoker: 11  On ART: 18  Integrase inhibitor: 13  Protease inhibitor: 5  Hydroxychloroquine: 14  Azithromycin: 12  Tocilizumab: 1  Experimental treatment: 2 |
| *Suwanwongse & Shabarek (34)* | 7 | 5 / 2 | 70+: 3  60-69: 1  50-59: 3  <50: 0 | N/A | Diabetes: 3  Hypertension: 5  Atrial fibrillation: 2  Heart failure: 1  Hepatitis C virus: 2  Chronic obstructive pulmonary disease: 4  Hyperlipidemia: 3 | Recent HIV RNA, <30: 2  Recent HIV RNA = 31: 1  Recent HIV RNA not detected: 3  Recent HIV RNA unknown: 1 | Recent CD4 count (mean): 645  Recent CD4 count values: 1827, 698, 243, 504, 179, 420 (1 unknown) | HAART compliance: 5  HAART non-compliance/not-taking: 2  Dyspnea: 5  Cough: 4  Fever: 3  Diarrhea: 1  Vomiting: 1  Bilateral ground glass opacities on CXR: 4  Bilateral interstitial infiltrates on CXR: 3  Hydroxychloroquine: 4  Antibiotics: 6  Mean length of stay in hospital (days): 7.86  Death due to septic shock from COVID-19: 3  Death due to ARDS from COVID-19: 2  Death due to hypoxemic RSF from COVID-19: 2 |
| *Tesoriero et al (35)* | 207 | 141 / 66 | 60+: 136  40 - <60: 67  <40: 4 | White, non-Hispanic: 20  Black, non-Hispanic: 85  Hispanic: 92  Other race: 10 | N/A | Virally suppressed at last test: 180  Not virally suppressed at last test: 21  Unknown if virally suppressed: 6 | N/A | Residence in Long Island, New York: 13  Residence in Mid-Hudson, New York: 5  Residence in New York City: 186  Residence in New York State, outside of New York City: 3  Heterosexual: 62  IDU: 55  MSM: 54  IDU & MSM: 7  In care, previous 12 months: 196 |
| *Venturas et al (36)* | 16 | 8 / 8 | Median: 47.5 | Black: 15  White: 0  Indian: 0  Mixed race: 1 | Cardiovascular disease: 7  Chronic kidney disease: 3  Chronic liver disease: 1  Chronic lung disease: 0  Diabetes mellitus: 7  Dyslipidaemia: 1  Thyroid disease: 0  Malignancy: 1  Obesity: 1  Tuberculosis: 1  Neuropsychiatric disease: 3 | Suppressed viral load: 4  Unsuppressed viral load: 5 | CD4 cells/mm^3^ (IQR): 180  CD4 >200 cells/mm^3^: 5 | Smoking: 4  Steroids: 13  ART: 11  Tocilizumab: 1  Length of stay: 9 |
